# Peer Review of “Toward Human Digital Twins for Cybersecurity Simulations on the Metaverse: Ontological and Network Science Approach”

**DOI:** 10.2196/38581

**Published:** 2022-04-20

**Authors:** Daniel Ayo Oladele

**Affiliations:** 1 Central University of Technology Bloemfontein South Africa

**Keywords:** human behavior modeling, cognitive twins, human digital twins, cybersecurity, cognitive systems, digital twins, Metaverse, artificial intelligence

*This is a peer-review report submitted for the paper “Toward Human Digital Twins for Cybersecurity Simulations on the Metaverse: Ontological and Network Science Approach.”*

## Round 1 Review

### General Comments

This paper [1] proposes a Cybonto conceptual framework for cybersecurity. It highlights the possibility of using human cognitive digital twin and digital twin systems for proactive cybersecurity strategies.

The paper was well written, the problem was clearly stated, the conceptual framework was well explained, and the author demonstrates an in-depth knowledge of cybersecurity ontologies, human cognitive digital twins, and behavioral or cognitive theories.

Looking forward to seeing the future works on this study.
